# The Complete Chloroplast Genome Sequences of 14 *Curcuma* Species: Insights Into Genome Evolution and Phylogenetic Relationships Within Zingiberales

**DOI:** 10.3389/fgene.2020.00802

**Published:** 2020-07-23

**Authors:** Heng Liang, Yan Zhang, Jiabin Deng, Gang Gao, Chunbang Ding, Li Zhang, Ruiwu Yang

**Affiliations:** ^1^College of Life Science, Sichuan Agricultural University, Yaan, China; ^2^School of Geography and Tourism, Guizhou Education University, Guiyang, China; ^3^College of Life Sciences and Food Engineering, Yibin University, Yibin, China; ^4^College of Science, Sichuan Agricultural University, Yaan, China

**Keywords:** Zingiberaceae, *Curcuma*, chloroplast genome, comparative analysis, phylogenetic analysis

## Abstract

Zingiberaceae is taxonomically complex family where species are perennial herb. However, lack of chloroplast genomic information severely hinders our understanding of Zingiberaceae species in the research of evolution and phylogenetic relationships. In this study, the complete chloroplast (cp) genomes of fourteen *Curcuma* species were assembled and characterized using next-generation sequencing. We compared the genome features, repeat sequences, sequence divergence, and constructed the phylogenetic relationships of the 25 Zingiberaceae species. In each Zingiberaceae species, the 25 complete chloroplast genomes ranging from 155,890 bp (*Zingiber spectabile*) to 164,101 bp (*Lanxangia tsaoko*) contained 111 genes consisting of 77 protein coding genes, 4 ribosomal RNAs and 30 transfer RNAs. These chloroplast genomes are similar to most angiosperm that consisted of a four-part circular DNA molecules. Moreover, the characteristics of the long repeats sequences and simple sequence repeats (SSRs) were found. Six divergent hotspots regions (*matK-trnk*, *Rps16-trnQ*, *petN-psbM*, *rpl32*, *ndhA*, and *ycf1*) were identified in the 25 Zingiberaceae chloroplast genomes, which could be potential molecular markers. In addition to *Wurfbainia longiligularis*, the ψ*ycf1* was discovered among the 25 Zingiberaceae species. The shared protein coding genes from 52 Zingiberales plants and four other family species as out groups were used to construct phylogenetic trees distinguished by maximum parsimony (MP), maximum likelihood (ML) and Bayesian inference (BI) and showed that Musaceae was the basal group in Zingiberales, and *Curcuma* had a close relationship with *Stahlianthu*. Besides this, *Curcuma flaviflora* was clustered together with *Zingiber*. Its distribution area (Southeast Asia) overlaps with the latter. Maybe hybridization occur in related groups within the same region. This may explain why Zingiberaceae species have a complex phylogeny, and more samples and genetic data were necessary to confirm their relationship. This study provide the reliable information and high-quality chloroplast genomes and genome resources for future Zingiberaceae research.

## Introduction

Over the past decade, due to the linkage of genetic material existed in the plastome, the whole plastid genomes have been used to assess the phylogenetic relationship and taxonomic significance in some areas. The chloroplast as one of crucial and essential organelle plays an irreplaceable role in several vital biochemical processes and photosynthesis of plant (Neuhaus and Emes, 2000). Chloroplast DNA has the characteristics of haploid inheritance, relatively small genome size and slow mutation rate in plants (Palmer et al., 1988). These chloroplast DNA features were used by scientists through comparing with chloroplast DNA phylogenies, proving to be very useful in understanding of plant phylogenetic studies and clearer taxonomic levels (Alwadani et al., 2019; Gonçalves et al., 2019). Based on previous research results, whole chloroplast genome was acceptably considered circular and its genes evolving was a single unit (Gitzendanner et al., 2018; Gu et al., 2019). Many recent studies have shown that the entire chloroplasts or protein-coding genes can provide stronger and greater phylogeography and phylogeny evidence (Shaw et al., 2014). The chloroplast DNA genome in complex genome plants has been proved comparatively inexpensive and easily sequenced (Guo et al., 2017; Saarela et al., 2018).

Although the chloroplast DNA genome data as a powerful and simple strategy has been used to analyze the evolutionary relationships of plant, their findings are discrepant and incongruous with the evidence of DNA barcoding makers and morphological character (Huang et al., 2014). Intergeneric or interspecific hybridization, gene flow, heterogeneity of evolutionary rates and sampling error, etc were the main sources of the discordance of different datasets (Rieseberg and Soltis, 1991). While these factors are not always a single one to cause discordance in the progress of phylogenies. Meanwhile, these factors also have different manifestations at different stages in the evolutionary processes operating therein (Joly et al., 2009; Kobayashi et al., 2017). The discrepant and incongruous evidence of DNA barcoding makers and morphological character have been widely used in explaining the problems of phylogeny (McKinnon et al., 1999; Meng and Kubatko, 2009). Thus, it is also a challenge to make sure the mechanism of inconformity. Different from the nuclear genes of amphilepsis, the chloroplast DNA is matrilineal inheritance and lacks recombination in most angiosperms. Therefore, chloroplast DNA datasets were used to reflect and analyze seed-mediated gene flow and reduce the influence of genes transmitted by pollen to confirm the female parent (Ruhlman et al., 2017). Compared to nuclear gene flow, chloroplast gene flow usually is easily identified (Du et al., 2009). Thus, the chloroplast gene is a useful evidence of interspecific gene flow.

Zingiberaceae, belonging to the order Zingiberales, is a controversial and complex (the classification and taxonomic status) flowering family of medicinal, edible and horticultural importance which widely occur in the regions of subtropical and tropical Asia (Soltis et al., 2000; Wood et al., 2000; Kress et al., 2002; Kanlayanapaphon and Newman, 2003). Due to lack of differences in morphological characters and a reliable reference genome, the phylogenetic relationships and compellent taxonomy of Zingiberaceae plants are still a huge challenge and intractable (Ye et al., 2008; Chen and Xia, 2010). Taking the genus *Curcuma* as an example, there are at least 120 species in the world, and about 10 *Curcuma* species are distributed in China (Li et al., 2001; Leong-Škorničková et al., 2007). *Curcuma* is a young genus and the hybridization and introgression are ubiquitous in its closely related species (Mallet, 2005). On account of the flower parts, shape and color of bracts, rhizome color and position of inflorescences in *Curcuma* plant are neither unique nor universal to all species; the traditional diagnostic characters identification of *Curcuma* is poor and difficult (Kress et al., 2002). In recent years, in order to deepen understanding of the taxonomy and phylogeny of *Curcuma*, the method based on modern branch was used by taxonomist and molecular phylogenetics (Deng et al., 2018).

However, use of only one or several certain DNA fragments usually leads to any additional evidence, incomplete conclusions and more confusion in Zingiberaceae species. Therefore, complete chloroplast genomes will be a better sequencing solutions to identifying species and reconstructing phylogeny relationship (Rogalski et al., 2015). Unfortunately, there are few publicly available chloroplast genomes data of Zingiberaceae species at present.

Similar studies were also performed on family or genus with taxonomic difficulties such as *Stahlianthus* (Li et al., 2019), *Alpinia* (Li et al., 2020), *Curcuma* (Gui et al., 2020), numerous previous endeavors have provided chloroplast genomes of 8 Zingiberaceae species and illuminated further insights into the phylogeny and taxonomy of the Zingiberaceae species (*Stahlianthus involucratus* clustered with *Curcuma longa* and *Curcuma roscoeana*, and *Alpinia* was a sister branch to *Amomum*), but still have not reached a satisfied resolution. Due to the incomplete cognition on Zingiberaceae classification, only few *Curcuma* species have often been described by different taxonomists. Here, we reported 14 new complete chloroplast genomes of *Curcuma* including seven firstly sequenced chloroplast genomes (*Curcuma zanthorrhiza, Curcuma elata, Curcuma yunnanensis, Curcuma alismatifolia, Curcuma amarissima, Curcuma sichuanensis*, and *Curcuma rosesana*) and combined with previously published Zingiberaceae complete chloroplast genomes data to visualize and test the genome organization, evolutionary rates, and phylogenetic relationships. All samples, assembled using a *de novo* approach, showed high similarities to previously published chloroplast genomes from *Curcuma*, including oligonucleotide repeats, microsatellites, RNA editing sites, amino acids frequencies, codon usage, synonymous and non-synonymous substitutions, GC content, gene synteny and genome sizes. Our goals were: (1) To observe the differences of the chloroplast genome in 25 Zingiberaceae species; (2) to find the highly polymorphic variable regions in chloroplast genomes and reliable markers for Zingiberaceae; (3) to infer the phylogenetic relationships within Zingiberaceae and discover the status of Zingiberaceae in Zingiberales based on the chloroplast genome.

## Results

### The Length and Features of Chloroplast Genome

**FIGURE 1:**
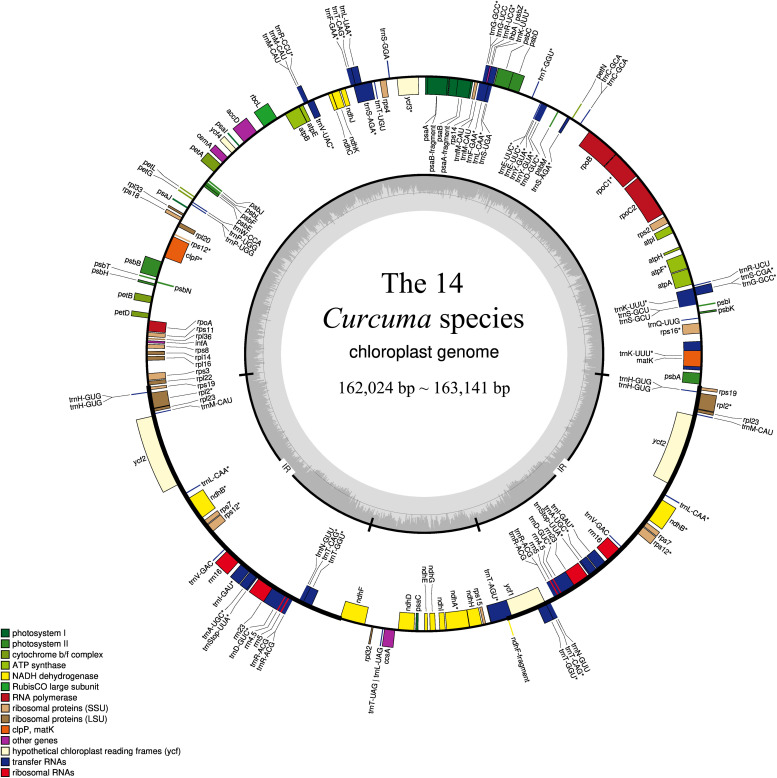
Gene structural map of the 14 *Curcuma* chloroplast genome. Genes of different functional groups, Small single copy (SSC), Large single copy (LSC), and Inverted repeats (IRa, IRb), are separated by color. Genes drawn inside the circle are transcribed clockwise.

The complete chloroplast genomes in the 25 Zingiberaceae species ranged in size from 155,890 bp (*Zingiber spectabile*) to 164,101 bp (*Lanxangia tsaoko*) (Table 1 and Supplementary Table S1). Similar to other studies of chloroplast genome, All the complete chloroplast genomes had a circular molecule conjoined and four conjoined structures (Xiang et al., 2016; Zhou et al., 2017). The quadripartite structure included the large single copy (LSC) region and small single copy (SSC) region separated by two inverted repeat (IR) regions (Figure 1). The LSC of the chloroplast genomes accounted for 53.58–58.27% of the total size and it ranged in size from 86,921 bp (*Curcuma wenyujin*) to 90,832 bp (*Zingiber spectabile*); the SSC of the chloroplast genomes accounted for 9.34–11.87% of the total size and it ranged in size from 152,88 bp (*Wurfbainia longiligularis*) to 185,08 bp (*Zingiber spectabile*). The IR of the chloroplast genomes accounted for 14.93–18.44% of the total size and it ranged in size from 30,119 bp (*Stahlianthus involucratus*) to 23,277 bp (*Zingiber spectabile*). In the genus *Curcuma*, the LSC of the chloroplast genomes accounted for 53.58–58.27% of the total size, ranging in size from 86,921bp (*Curcuma wenyujin*) to 87,856 bp (*Curcuma flaviflora*); the SSC of the chloroplast genomes accounted for 9.53–9.68% of the total size, ranging in size from 15,372 bp (*Curcuma alismatifolia*) to 15,744 bp (*Curcuma flaviflora*). The IR of the chloroplast genomes accounted for 18.26–18.37% of the total size and ranging in size from 29,718 bp (*Curcuma wenyujin*) to 29,897 bp (*Curcuma alismatifolia*). The GC contents of the chloroplast genomes in the family Zingiberaceae were similar.

**TABLE 1:** Complete chloroplast genomes for 14 Curcuma species.

	***C. rosesana***	***C. amarissima***	***C. sichuanensis***	***C. elata***	***C. zanthorrhiza***	***C. longa***	***C. yunnanensis***
Total length (bp)	162157	162158	162133	162171	162192	162220	162133
GC (%)	36.21	36.2	36.22	36.22	36.2	36.19	36.22
**LSC**							
Length (bp)	87043	86982	87013	87037	86994	87041	87013
GC (%)	33.98	34	34	34.01	33.99	33.99	34
Length (%)	53.68	53.64	53.67	53.67	53.64	53.66	53.67
**SSC**							
Length (bp)	15616	15678	15622	15634	15700	15681	15622
GC (%)	29.75	29.65	29.77	29.76	29.66	29.65	29.77
Length (%)	9.63	9.67	9.64	9.64	9.68	9.67	9.64
**IR**							
Length (bp)	29751	29751	29751	29752	29751	29751	29751
GC (%)	41.14	41.15	41.15	41.15	41.15	41.14	41.15
Length (%)	18.35	18.35	18.35	18.35	18.34	18.34	18.35

	***C. sp.1***	***C. sp.2***	***C. wenyujin***	***C. phaeocaulis***	***C. aromatica***	***C. alismatifolia***	***C. flaviflora***

Total length (bp)	162155	162133	162024	162031	162243	162715	163141
GC (%)	36.21	36.22	36.22	36.22	36.21	36.15	36.09
**LSC**							
Length (bp)	87041	87013	86921	86923	87109	87416	87856
GC (%)	33.99	34	34.03	34.03	33.99	33.91	33.85
Length (%)	53.68	53.67	53.65	53.65	53.69	53.72	53.85
**SSC**							
Length (bp)	15616	15622	15671	15676	15634	15509	15744
GC (%)	29.75	29.77	29.72	29.71	29.76	29.82	29.52
Length (%)	9.63	9.64	9.67	9.67	9.64	9.53	9.65
**IR**							
Length (bp)	29751	29751	29718	29718	29752	29897	29784
GC (%)	41.14	41.15	41.14	41.14	41.15	41.05	41.12
Length (%)	18.35	18.35	18.34	18.34	18.34	18.37	18.26

*GC, guanine-cytosine; LSC, large single-copy region; SSC, short single-copy region; IRs, inverted repeats.*

Among the 111 functional genes, there are 77 protein-coding genes, 30 tRNA genes and 4 rRNA genes in the Zingiberaceae plastomes encoded (Table 2), quite similar to previously published Zingiberaceae species. Among these chloroplast genes of 25 Zingiberaceae species, 54 gene fragments were connected with self-replication, 4 genes were connected with DNA dependent RNA polymerase, 8 genes were connected with large subunit of ribosome, 12 genes were connected with small subunit of ribosome, 44 genes were connected with photosynthesis, 5 genes were connected with encoded the subunits of photo system I, 6 genes were connected with ATP synthesis, 14 genes were connected with encoded the subunits of photo system II, 11 genes were connected with subunits of NADH-dehydrogenase, 6 genes were connected with subunits of cytochrome, and only one gene was connected with subunits of rubisco. The *infA* gene was related to the translational initiation factor. The protein coding exons accounted for 47.29–52.18% of the total length and regions ranged from 77,372 to 84,187 bp, the rRNA accounted for 5.52–5.81% of the total length and regions ranged from 9,054 to 9,102 bp, the tRNA accounted for 1.41–1.87% of the total length and regions ranged from 2,311 to 3,029 bp, the intergenic regions accounted for 29.58–38.11% of the total length and ranged from 47,943 to 62,355 bp, the intronic regions accounted for 7.59–13.87% of the total length and ranged from 12,416 to 22,757 bp (Supplementary Table S2). In the genus *Curcuma*, the protein coding exons accounted for 50.83–51.19% of the total length and regions ranged from 82,930 to 83,042 bp, the rRNA accounted for 5.55–5.59% of the total length and regions ranged from 9,054 to 9,058 bp, the tRNA accounted for 1.79–1.80% of the total length and regions were 2,920 bp, the intergenic regions accounted for 29.58%–30.00% of the total length and ranged from 47,943 to 48,949 bp, the intronic regions ranged accounted for 11.82–11.90% of the total length and ranged from 19,164 to 19,364 bp (Table 3). Introns were more easy to lose its function than excons during evolution (Mattick, 1994; Kelchner, 2002; Chorev and Carmel, 2012). Among the 111 distinct genes, 18 genes were connected to contained introns. The genes of *ycf3* and *clpP* contained two introns; a total of 14 genes contained one intron, including 8 coding genes and 6 tRNA genes. In most Zingiberaceae species, a total of 18 genes were related to introns. Among the 18 genes, there were13 genes in the LSC regions, 4 genes in the IR regions and only one gene in the SSC regions (Supplementary Table S3).

**TABLE 2:** Genes contained in the chloroplast genome sequence of 25 Zingiberaceae species.

**Gene category**	**Groups of genes**	**Name of genes**
Self-replication	Ribosomal RNAs	*rrn16^b^;rrn23^b^;rrn4.5^b^;rrn5^b^*
	Transfer RNAs	*trnA-UGC^a,b^*; *trnC-GCA*; *trnD-GUC*; *trnE-UUC*; *trnF-GAA*; *trnfM-CAU trnG-UCC^a^*; *trnG-GCC*; *trnH-GUG*; *trnI-CAU^b^ trnI-GAU^a,b^*; *trnK-UUU^a^ trnL-CAA^b^*; *trnL-UAA^a^*; *trnL-UAG*; *trnM-CAU*; *trnN-GUU^b^*; *trnP-UGG trnQ-UUG*; *trnR-ACG^b^*; *trnR-UCU*; *trnS-GCU*; *trnS-GGA*; *trnS-UGA trnT-GGU*; *trnT-UGU*; *trnV-GAC*; *trnV-UAC^a^; trnW-CCA*;*trnY-GUA*
	Small subunit of ribosome	*rps2*; *rps3*; *rps4*; *rps7*^b^; *rps8*; *rps11*; *rps12*^a,b^; *rps14*; *rps15*; *rps16*^a^; *rps18*; *rps19*
	Large subunit of ribosome	*rpl2*^a,b^; *rpl14*; *rpl16*^a^; *rpl20*; *rpl23*^b^; *rpl32*; *rpl33*; *rpl36*
	DNA dependent RNA polymerase	*rpoA*; *rpoB*; *rpoC1*^a^; *rpoC2*
Photosynthesis	Subunits of photosystem I	*psaA*; *psaB*; *psaC*; *psaI*; *psaJ*
	Subunits of photosystem II	*psbA*; *psbB*; *psbC*; *psbD*; *psbE*; *psbF*; *psbI; psbJ*; *psbK; psbL*; *psbM*; *psbN*; *psbT*; *psbZ*
	Subunits of cytochrome	*petA*; *petB*^a^; *petD*; *petG*; *petL*; *petN*
	Subunits of ATP synthase	*atpA*; *atpB*; *atpE*; *atpF*^a^; *atpH*; *atpI*
	ATP-dependent protease subunit p gene	*clpP*^a^
	Large subunit of Rubisco	*rbcL*
	Subunits of NADH dehydrogenase	*ndhA*^a^; *ndhB*^a,b^; *ndhC*; *ndhD*; *ndhE*; *ndhF*; *ndhG*; *ndhH*; *ndhI*; *ndhJ*; *ndhK*
Other genes	Maturase	*matK*
	Envelop membrane protein	*cemA*
	Acetyl-CoAcarboxylase	*accD*
	c-type cytochrom synthesis gene	*ccsA*
	Translational initiation factor	*infA*
Genes of unknown function	Conserved open reading frames	*ycf1*^b^; *ycf2*^b^; *ycf3*^a^; *ycf4*

*^*a*^Intron-containing genes. ^*b*^Genes located in the IR regions.*

**TABLE 3:** Distribution of genes and intergenic regions for 14 species in *Curcuma.*

	***C. rosesana***	***C. amarissima***	***C. sichuanensis***	***C. elata***	***C. zanthorrhiza***	***C. longa***	***C. yunnanensis***
Protein Coding Genes Length (bp)	82975	82975	82975	82975	82975	82975	82975
GC (%)	36.91	36.92	36.92	36.92	36.92	36.91	36.92
Length (%)	51.17	51.17	51.18	51.17	51.16	51.15	51.18
**rRNA**							
Length (bp)	9054	9054	9054	9054	9054	9054	9054
GC (%)	55.15	55.15	55.15	55.15	55.15	55.15	55.15
Length (%)	5.58	5.58	5.58	5.58	5.58	5.58	5.58
**tRNA**							
Length (bp)	2920	2920	2920	2920	2920	2920	2920
GC (%)	52.91	52.91	52.91	52.91	52.91	52.91	52.91
Length (%)	1.8	1.8	1.8	1.8	1.8	1.8	1.8
**Intergenic Regions**							
Length (bp)	47992	47997	47967	47979	48019	48055	47967
GC (%)	30.19	30.17	30.22	30.23	30.15	30.16	30.22
Length (%)	29.6	29.6	29.58	29.59	29.61	29.62	29.58
**Intron**							
Length (bp)	19216	19212	19217	19243	19224	19216	19217
GC (%)	19216	19212	19217	19243	19224	19216	19217
Length (%)	11.85	11.85	11.85	11.87	11.85	11.85	11.85

	***C. sp.1***	***C. sp.2***	***C. wenyujin***	***C. phaeocaulis***	***C. aromatica***	***C. alismatifolia***	***C. flaviflora***

Protein Coding Genes Length (bp)	82975	82975	82943	82943	82975	83042	82930
GC (%)	36.91	36.92	36.94	36.94	36.92	36.9	36.89
Length (%)	51.17	51.18	51.19	51.19	51.14	51.04	50.83
**rRNA**							
Length (bp)	9054	9054	9054	9054	9054	9058	9054
GC (%)	55.15	55.15	55.15	55.15	55.15	55.17	55.15
Length (%)	5.58	5.58	5.59	5.59	5.58	5.57	5.55
**tRNA**							
Length (bp)	2920	2920	2920	2920	2920	2920	2920
GC (%)	52.91	52.91	52.91	52.91	52.91	52.88	52.95
Length (%)	1.8	1.8	1.8	1.8	1.8	1.79	1.79
**Intergenic Regions**							
Length (bp)	47990	47967	47943	47947	48051	48331	48949
GC (%)	30.19	30.22	30.22	30.22	30.21	30.11	29.97
Length (%)	29.6	29.58	29.59	29.59	29.62	29.7	30
**Intron**							
Length (bp)	19216	19217	19164	19167	19243	19364	19288
GC (%)	19216	19217	19164	19167	19243	19364	19288
Length (%)	11.85	11.85	11.83	11.83	11.86	11.9	11.82

*GC, guanine-cytosine; LSC, large single-copy region; SSC, short single-copy region; IRs, inverted repeats.*

### Codon Usage

The codon usage frequency of 64 coding genes for 25 Zingiberaceae species were estimated. All protein-coding genes were encoded by 37,160 (*Amomum krervanh*) to 52,160 (*Wurfbainia villosa*). UGA, UAG, and UAA were considered as the termination codons. For these Zingiberaceae species (Table 4 and Supplementary Table S4), we found that the UUA encoded leucine had the highest RSCU (Relative Synonymous Codon Usage) value at approximately 1.92, and the GCG encoded alanine had the lowest RSCU value at approximately 0.33. The AAA encoded lysine was the most frequent amino acid and had more than 2,200 frequencies in most of the 25 Zingiberaceae species. The results of RSCU in the 25 Zingiberaceae species showed G or C was biased toward a lower nucleotide frequency than A or T at the third codon position and the result was similar to other angiosperms chloroplast genomes researches.

**TABLE 4:** Codon content of amino acid and stop codon of 8 species.

		***C. sichuanensis***	***A. compactum***	***A. oxyphylla***	***W. villosa***	***Z. officinale***	***K. galanga***	***S. involucratus***	***L. tsaoko***
**Amino acid**	**Codon**				**RSCU^a^**				
Ala	GCA	1.31	1.29	1.29	1.29	1.25	1.29	1.32	1.29
Ala	GCC	0.56	0.54	0.55	0.55	0.58	0.56	0.55	0.56
Ala	GCG	0.32	0.34	0.31	0.34	0.32	0.32	0.32	0.33
Ala	GCU	1.81	1.83	1.85	1.83	1.86	1.83	1.81	1.82
Cys	UGC	0.54	0.55	0.53	0.55	0.5	0.54	0.56	0.55
Cys	UGU	1.45	1.45	1.47	1.45	1.5	1.46	1.44	1.45
Asp	GAC	0.35	0.37	0.38	0.37	0.36	0.37	0.36	0.38
Asp	GAU	1.65	1.63	1.62	1.63	1.64	1.63	1.64	1.62
Glu	GAA	1.49	1.5	1.52	1.5	1.49	1.51	1.5	1.51
Glu	GAG	0.51	0.5	0.48	0.5	0.51	0.49	0.51	0.49
Phe	UUC	0.71	0.7	0.68	0.71	0.71	0.69	0.71	0.7
Phe	UUU	1.29	1.3	1.32	1.29	1.29	1.31	1.29	1.3
Gly	GGA	1.59	1.61	1.6	1.59	1.59	1.6	1.6	1.59
Gly	GGC	0.34	0.33	0.33	0.34	0.36	0.36	0.34	0.34
Gly	GGG	0.67	0.65	0.63	0.67	0.61	0.62	0.67	0.68
Gly	GGU	1.4	1.41	1.44	1.4	1.43	1.42	1.39	1.39
His	CAC	0.43	0.42	0.41	0.42	0.45	0.44	0.44	0.43
His	CAU	1.57	1.58	1.59	1.58	1.55	1.56	1.56	1.57
Ile	AUA	0.97	0.96	0.98	0.97	0.96	0.98	0.97	0.99
Ile	AUC	0.55	0.55	0.53	0.55	0.53	0.53	0.56	0.54
Ile	AUU	1.48	1.48	1.49	1.48	1.51	1.49	1.47	1.48
Lys	AAA	1.47	1.47	1.47	1.47	1.5	1.5	1.47	1.43
Lys	AAG	0.53	0.53	0.53	0.53	0.5	0.5	0.53	0.57
Leu	CUA	0.8	0.79	0.8	0.8	0.76	0.8	0.8	0.84
Leu	CUC	0.41	0.41	0.41	0.41	0.4	0.39	0.4	0.4
Leu	CUG	0.38	0.38	0.36	0.37	0.37	0.36	0.4	0.38
Leu	CUU	1.24	1.24	1.24	1.26	1.23	1.23	1.24	1.27
Leu	UUA	1.86	1.89	1.93	1.88	1.94	1.95	1.84	1.83
Leu	UUG	1.31	1.3	1.26	1.29	1.3	1.25	1.31	1.28
Met	AUG	1	1	1	1	1	1	1	1
Asn	AAC	0.47	0.46	0.46	0.46	0.45	0.47	0.48	0.48
Asn	AAU	1.53	1.54	1.54	1.54	1.55	1.53	1.52	1.52
Pro	CCA	1.18	1.2	1.19	1.18	1.18	1.15	1.18	1.19
Pro	CCC	0.73	0.75	0.77	0.74	0.77	0.76	0.73	0.73
Pro	CCG	0.48	0.47	0.44	0.47	0.48	0.49	0.48	0.48
Pro	CCU	1.62	1.58	1.6	1.6	1.57	1.6	1.61	1.59
Gln	CAA	1.52	1.51	1.52	1.51	1.51	1.52	1.52	1.52
Gln	CAG	0.48	0.49	0.48	0.49	0.49	0.48	0.48	0.48
Arg	AGA	1.9	1.88	1.89	1.9	1.94	1.88	1.87	1.93
Arg	AGG	0.75	0.74	0.7	0.73	0.71	0.7	0.78	0.75
Arg	CGA	1.25	1.27	1.27	1.27	1.24	1.29	1.24	1.24
Arg	CGC	0.35	0.34	0.33	0.34	0.34	0.35	0.34	0.35
Arg	CGG	0.46	0.47	0.42	0.47	0.41	0.42	0.47	0.45
Arg	CGU	1.3	1.3	1.38	1.28	1.36	1.36	1.3	1.28
Ser	AGC	0.35	0.35	0.36	0.35	0.35	0.35	0.36	0.36
Ser	AGU	1.23	1.22	1.3	1.22	1.34	1.31	1.22	1.2
Ser	UCA	1.18	1.15	1.15	1.16	1.16	1.17	1.17	1.19
Ser	UCC	0.99	0.99	0.95	0.97	0.97	0.95	1	0.97
Ser	UCG	0.55	0.58	0.54	0.57	0.54	0.53	0.56	0.59
Ser	UCU	1.69	1.71	1.7	1.72	1.64	1.69	1.69	1.7
Thr	ACA	1.28	1.28	1.28	1.28	1.25	1.29	1.27	1.28
Thr	ACC	0.72	0.69	0.69	0.7	0.67	0.69	0.71	0.7
Thr	ACG	0.47	0.46	0.45	0.47	0.43	0.44	0.46	0.46
Thr	ACU	1.54	1.58	1.58	1.55	1.64	1.58	1.56	1.57
Val	GUA	1.48	1.51	1.49	1.49	1.5	1.48	1.5	1.47
Val	GUC	0.49	0.49	0.45	0.49	0.48	0.47	0.5	0.48
Val	GUG	0.57	0.55	0.57	0.57	0.56	0.56	0.57	0.58
Val	GUU	1.46	1.45	1.49	1.45	1.46	1.49	1.44	1.46
Trp	UGG	1	1	1	1	1	1	1	1
Tyr	UAC	0.44	0.43	0.43	0.43	0.43	0.44	0.43	0.44
Tyr	UAU	1.56	1.57	1.57	1.57	1.57	1.56	1.57	1.56
Stop*	UAA	1.28	1.23	1.3	1.28	1.43	1.33	1.26	1.22
Stop*	UAG	0.91	0.94	0.91	0.93	0.83	0.92	0.9	1
Stop*	UGA	0.81	0.83	0.79	0.8	0.75	0.76	0.83	0.78

^*a*^Relative synonymous codon usage; * Stop codon.

### Comparative Chloroplast Genomic Analysis Within 25 Zingiberaceae Species

**FIGURE 2:**
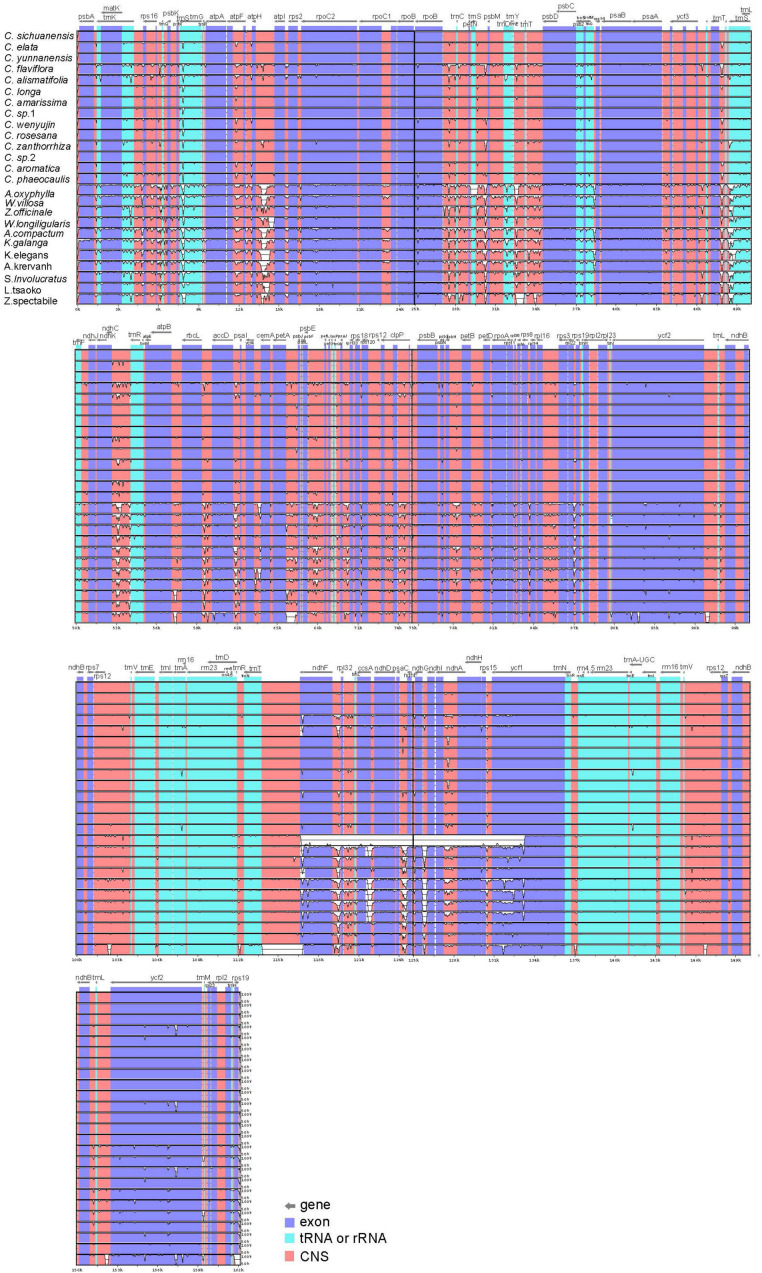
Sequence alignment of 25 Zingiberaceae chloroplast genome with *C. sichuanensis* as a reference by using in mVISTA. The Y-scale represents the percentage of identity ranging from 50 to 100%.

To characterize genome divergence, we used the program mVISTA to perform sequence alignments of 25 Zingiberaceae species and took the annotation of *Curcuma sichuanensis* as a reference. The comparison indicated that the 25 chloroplast genomes were highly similar (Figure 2). The characterize genome divergence was low among the 14 *Curcuma* species. The results indicated the divergence in IR regions was lower than in LSC and SSC regions. Moreover, the variation of divergence in coding regions was less than that in non-coding. Among the coding genes, the highly divergent regions included *matK*, *rpoC2*, *rpoB*, *ndhF*, *ycf1*, and *ycf2*. In addition, some of the highly divergent regions, such as *psbK-psbI*, *atpH-atplI*, *rpoB-petN*, *trnS-psbM*, *trnT-psbD*, *ndhC-trnR*, *petA-psbJ*, and *ndhF-rpl32*, were among the intergenic regions.

The number and sequence of genes in the IR regions were more conserved than that in the LSC and SSC regions within the 25 chloroplast genomes. The IR amplification and contraction were the causes of the differences of chloroplast genome size among the 25 Zingiberaceae species. Among the analysis of the genes across 25 chloroplast genomes, it is obvious that the evolutionary rates had some differences. In summary, the average number of Ka/Ks was less than 0.5 for 92.54% genes. The 20 genes showed Ka/Ks > 1 in at least one species. There were two genes (*ndhc* and *rps8*) showing a high rates for more than half species, and the genes of *ndhc* and *rps8* may be under positive selection. Most of genes associated with photosynthesis showed the lowest rates of evolution, and seven of them (*atpH*, *petL*, *petN*, *psaC*, *psaJ*, *psbM*, and *psbT*) showed Ka/Ks were 0. The Ka/Ks of *atpH*, *petL*, *petN*, *psaC*, *psaJ*, *psbM*, *psbT*, *rps2*, and *rps7* were low, and the main reason was no non-synonymous substitutions (Supplementary Table S5). We also inferred three genes with the most variable Ka/Ks, *atpF* (Subunits of ATP synthase), *rps8* (Small subunit of ribosome) and *ndhC* (Subunits of NADH dehydrogenase). For the *atpF* gene, the values of Ka/Ks were 0.2397 to 1.3544 (aside from a few Ka were 0), the *rps8* gene ranged from 0.1290 to 1.0608 and the *ndhC* gene ranged from 0.1593 to 1.3253. The result indicated that the genes of *ndhC* and *rps8* evolved slower than *atpF* gene.

### The Differences of Genome Size Among the 25 Zingiberaceae Species

Among the 25 Zingiberaceae species, the shortest genome was *Zingiber spectabile* (155,890 bp), and the longest was *Lanxangia tsaoko* (164,101). In the 14 *Curcuma* species, the genome length of *Curcuma flaviflora* was the longest (163,141 bp) while that of *Curcuma wenyujin* was the shortest (162,024 bp). Except for *Curcuma flaviflora* and *Curcuma alismatifolia*, the sizes of the chloroplast genomes of *Curcuma* species were between 162,024 bp and 162,243 bp (Table 1). Except *Zingiber spectabile*, lengths of other genera of Zingiberaceae species were greater than 160,000 bp (Supplementary Table S1). In a word, the sizes of the chloroplast genomes of 14 *Curcuma* species were significantly similar to those of other Zingiberaceae species. In the 25 Zingiberaceae species, the sizes of the intergenic regions (IGS) ranged from 47,943 to 62,355 bp, and the sizes of the IGS ranged from 47,943 to 48,949 bp among the 14 *Curcuma* species, similar to the sizes of the complete chloroplast genomes (Table 3 and Supplementary Table S2). As most same angiosperm plants, the difference of the variation in genome sizes was caused by the size of IGS. In the 25 Zingiberaceae species, the percentage of GC content was 36.00–36.29% with an average of 36.04%. In the 14 *Curcuma* species, the percentage of GC content was 36.09–36.22% (an average of 36.20%), which was higher than that of the other genera of Zingiberaceae species (36.09%) (Table 1 and Supplementary Table S1).

### Contraction and Expansion of Inverted Repeats (IRs) Among the 25 Zingiberaceae Species

**FIGURE 3:**
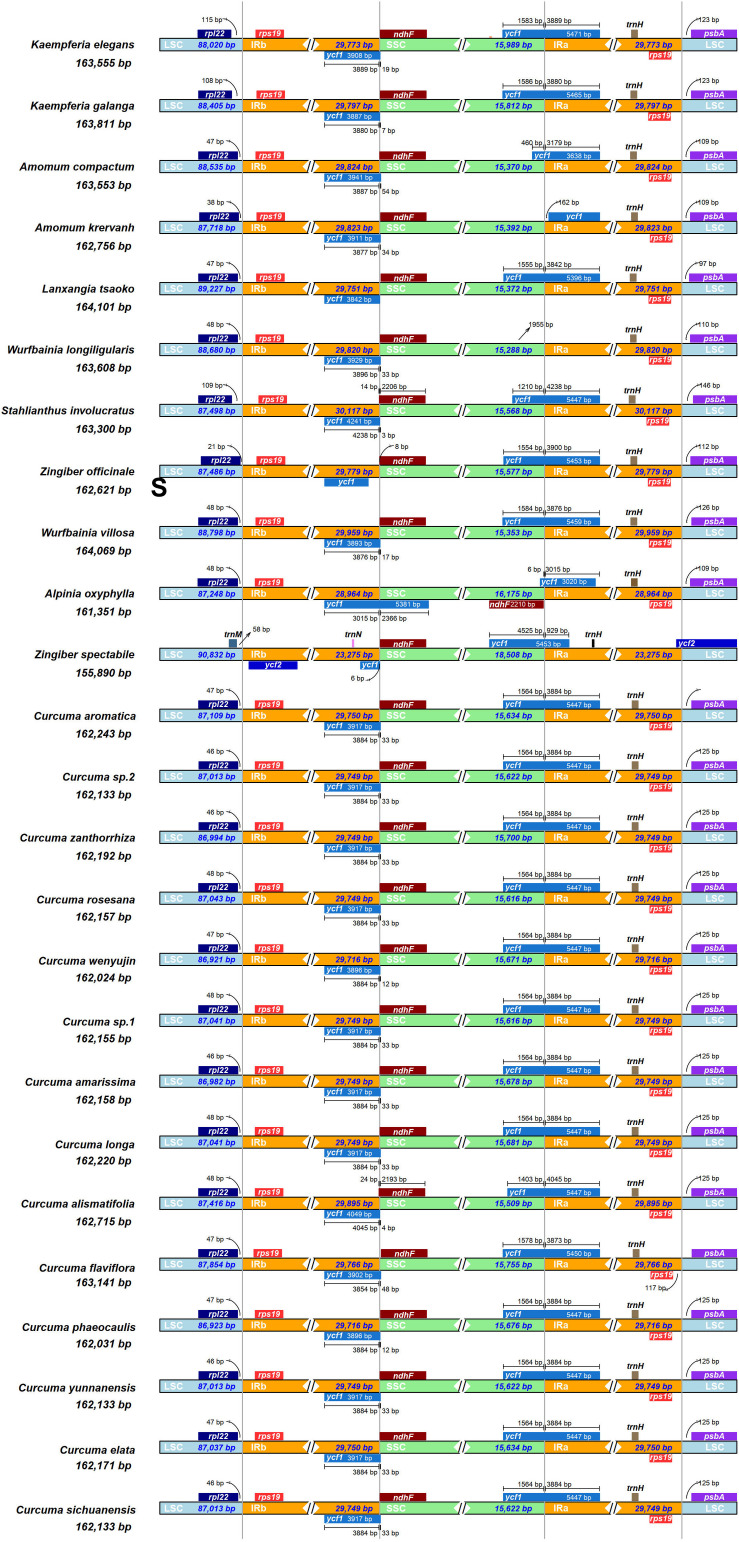
Comparison of the junctions between the LSC, SSC and IR regions among 25 Zingiberaceae chloroplast genome. The figure is not to scale LSC, SSC, and IR.

Of the 25 Zingiberaceae species, the genomic structure was highly conserved, including the number and sequence of genes. But, the IRa and IRb boundaries changed in structure (Figure 3). Although the sizes of the IR region were more highly conserved than the other regions, and the contraction and expansion of IR boundaries were accounted as playing a valuable role in genome size (Gu et al., 2017).

Among the 25 Zingiberaceae species, the sizes of the IRs of *Stahlianthus involucratus* was the longest (30,119 bp) and that of *Zingiber spectabile* was the shortest (23,277 bp). In the 14 *Curcuma* species, the sizes of the IRs of *Curcuma flaviflora* was the longest (29,784 bp) and that of *Curcuma elata* was the shortest (29,752 bp). Within the 25 Zingiberaceae species, the *rpl22*-*rps19* genes were located in the boundaries of LSC/IRb regions except *Zingiber spectabile*, in which there were *trnM-ycf2* genes and the *rpl22*-*rps19* gene was missing in the junctions of the LSC/IRb regions. The *ycf1*-*ndhF* genes were located at the IRb/SSC boundary of 25 Zingiberaceae species. Except *Alpinia oxyphylla*, IRb/SSC boundary was embedded in the *ndhF* genes. The IRb/SSC and SSC/IRa regions were variable. Except *Amomum krervanh*, *Wurfbainia longiligularis* and *Alpinia oxyphylla*, the SSC/IRa junctions in the chloroplast genomes of Zingiberaceae species was embedded in the *ycf1* gene, with 929–4,238 bp in the IRa region. The *rps19*-*psbA* genes were located in the junctions of IRa/LSC regions in the 24 Zingiberaceae species except *Zingiber spectabile*, in which the *ycf2* genes was located in the junctions of IRa/LSC regions, while the *rps19*-*psbA* genes could not be found in the junctions of IRa/LSC regions. For the 14 *Curcuma* species, *psbA* was located on the right side of IRa/LSC regions with the same distance of 25 bp. For the 10 Zingiberaceae species (*Amomum krervanh*, *Amomum compactum*, *Wurfbainia longiligularis*, *Wurfbainia villosa*, *Kaempferia elegans*, *Kaempferia galangal*, *Alpinia oxyphylla*, *Stahlianthus Involucratus*, *Lanxangia tsaoko*, and *Zingiber officinale*), *psbA* was located on the right side of IRa/LSC regions with the distance of 97–146 bp. In the IRb region of the 25 Zingiberaceae species, we found the *rps19* was pseudogene, and the reason was that the *rps19* coding gene existed in the boundaries of IRa-LSC. In summary, the contraction and expansion of inverted repeats were detected in the 25 Zingiberaceae species.

### Repeat Structure Analysis

**FIGURE 4:**
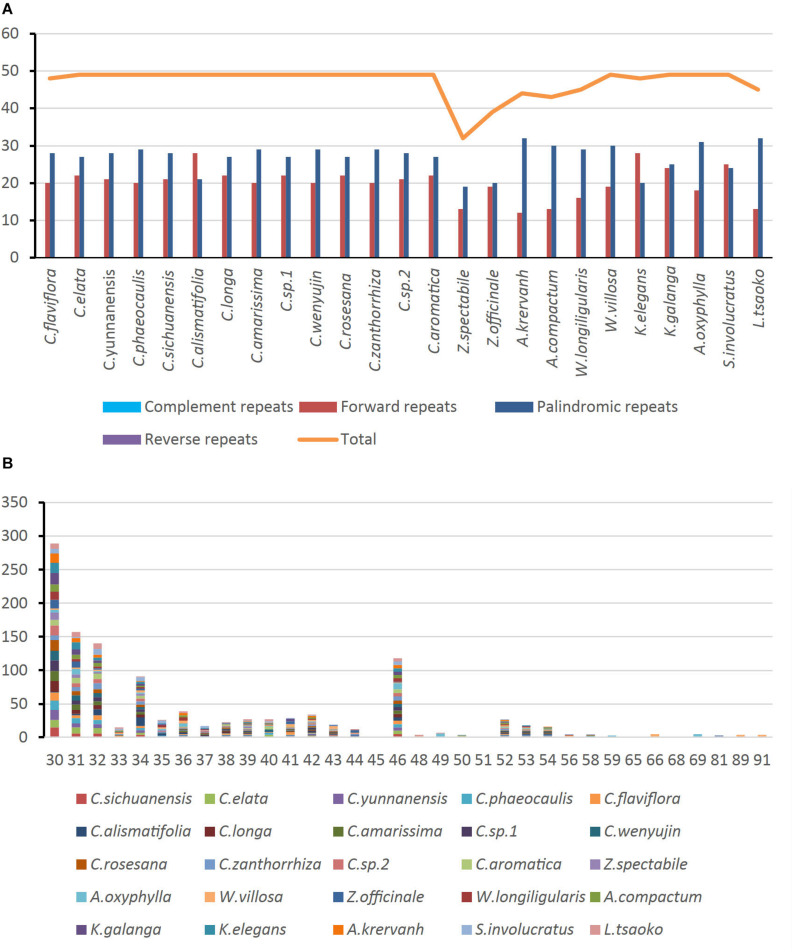
Number of long repetitive repeats on the complete chloroplast genome sequence of 25 Zingiberaceae species; **(A)** frequency of repeat type; **(B)** frequency of the repeats.

Of the 25 Zingiberaceae species, there were a total of 1,177 long repeats of two types (Supplementary Table S6). Among these Zingiberaceae species, all of them had forward and palindromic repeats. Except *Curcuma flaviflora*, the other species of the *Curcuma* had the same number of long repeats. For the other Zingiberaceae species, the number of repeats were 32–49. Most of Zingiberaceae species had 49 long repeats. *Zingiber spectabile* which had the smallest number of repeats had 19 palindromic and 13 forward repeats (Figure 4A). On the whole, the range of copy length was 30–91 bp. The numbers of most repeat sequences in the total length were 30, 31, 32, and 46 (Figure 4B).

### Simple Sequence Repeat (SSR) Analysis

**FIGURE 5:**
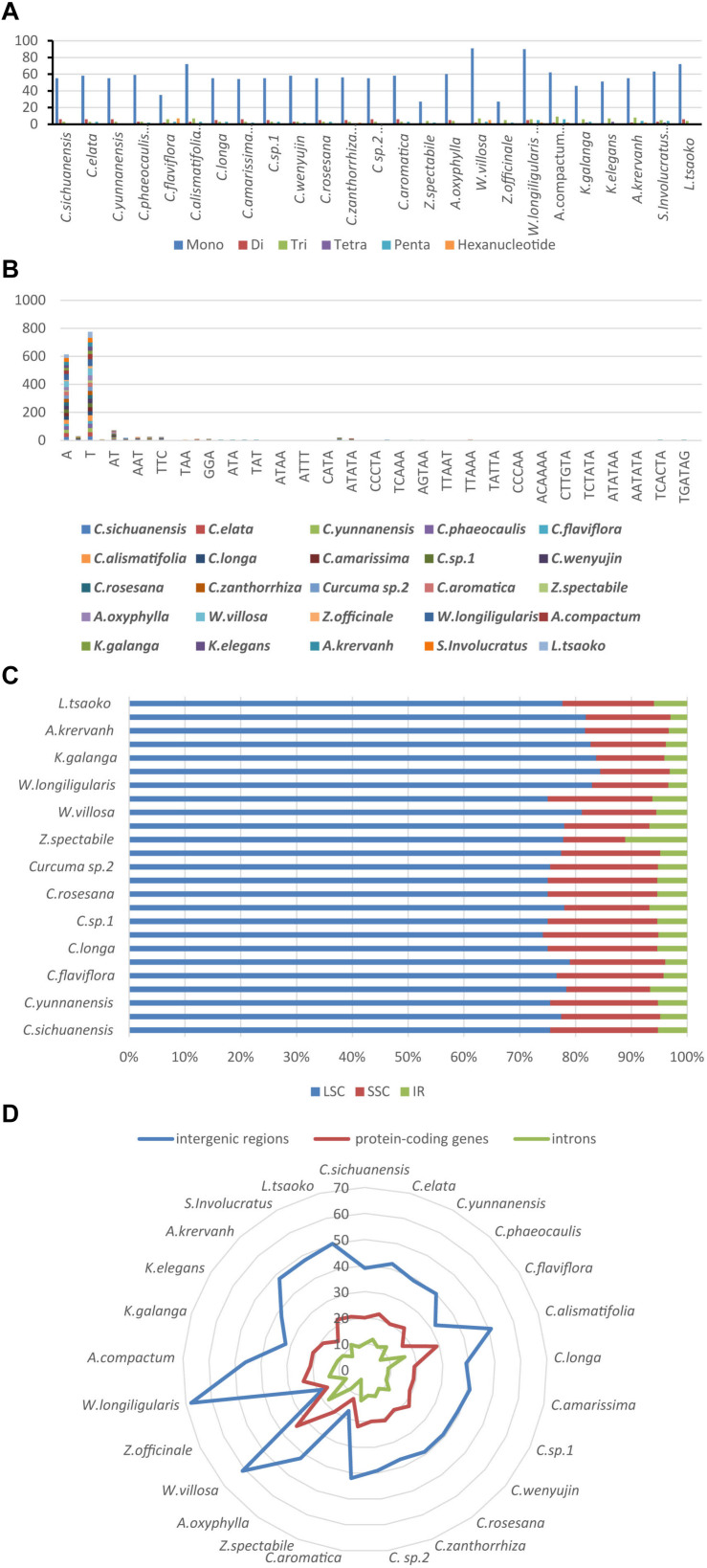
The comparison of SSR distribution in 25 chloroplast genomes; **(A)** number of different SSR types; **(B)** frequency of common motifs; **(C)** frequency of SSRs in the LSC, IR, SSC region; **(D)** frequency of SSRs in the intergenic regions, protein-coding genes and introns.

Simple sequence repeats (SSRs), also called microsatellites, are made up of short and tandem repeat nucleotide sequences with sizes of 1–6 bp (Powell et al., 1995). SSRs are widely distributed in the chloroplast genome, and play a critical role in species authentication and are applied to molecular markers (Doorduin et al., 2011; Park et al., 2016; Yan et al., 2019). Here, we found that the distribution of 33–108 SSRs in the Zingiberaceae species that ranged in size from 10 to 20 bp (Figure 5 and Supplementary Table S7). There were 6 kinds of SSRs that were discovered. Among these SSRs, only the chloroplast genomes of *Curcuma flaviflora*, *Curcuma zanthorrhiza*, *Wurfbainia villosa*, *Wurfbainia longiligularis*, *Amomum compactum*, *Amomum krervanh* and *Kaempferia galangal* had the hexa-nucleotide repeats, and the highest number of hexa-nucleotide repeats was detected in *Curcuma flaviflora*. Among 25 Zingiberaceae species, the numbers of mononucleotide repeats ranged from 27 to 90, followed by trinucleotide ranging from 3 to 9; pentanucleotide ranging from 6 to 1; hexa-nucleotide ranging from 0 to 7; dinucleotide ranging from 0 to 6 and tetranucleotide ranging from 0 to 3 (Figure 5A). Therefore, mononucleotide repeats may play a more significant role than the other SSRs in genetic variation (Jiao et al., 2012).

Among these SSRs in the 25 Zingiberaceae species, the percentages of A/T mononucleotide repeats were 43.12 and 54.35% respectively. The percentages of C/G mononucleotide repeats were 2.1 and 0.43% respectively. The combination of A/T in most the other SSRs may be the reason for bringing about the high AT content in the whole chloroplast genomes within the 25 Zingiberaceae species (Figure 5B). Among all SSR types, previous studies found that A/T always richest continually used bases (Wu et al., 2017). In the 14 *Curcuma* species, the number of A mononucleotide repeats ranged from 24 to 30, with T mononucleotide repeats ranging from 28 to 41, except in *Curcuma flaviflora*. In addition, in the same genus, we can infer that the number of A/T repeats may be similarity. However, in *Curcuma flaviflora* was 15/20, which were much lower than those of the other 13 *Curcuma* species. This may be the main reason why the lower intergenic spacers were in *Curcuma flaviflora*.

Among the 25 Zingiberaceae species (Figure 5C), SSRs were located in intergenic spacers, with an average of 41. The next SSRs were distributed in coding genes with an average of 20. The least number of SSRs were found in the introns, with an average of 10 (Figure 5D). The SSR loci were located in 5 coding genes (*rpoC2*, *rps14*, *petA*, *rps18*, and *ycf1)* and 32 intergenic regions of the 25 Zingiberaceae species. Therefore, these results suggest that the availability of SSRs can be greatly applied to develop useful molecular markers for identifying genetic diversity, evolutionary studies and phylogeny.

### Divergence Hotspots

**FIGURE 6:**
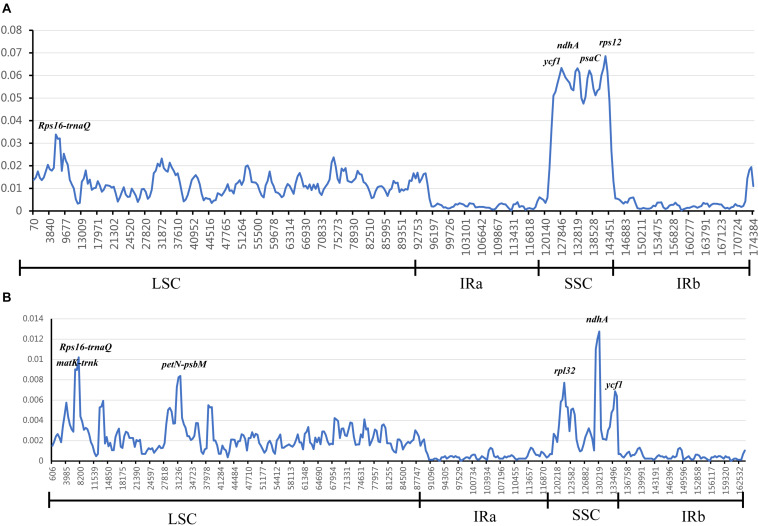
Sliding window analysis of 25 Zingiberaceae chloroplast genomes. The nucleotide variability (Pi) value in: **(A)** 25 Zingiberaceae species; **(B)** 14 *Curcuma* species.

In chloroplast genomes research, divergent hotspots are usually used as important evidence for species authentication and provide information about phylogeny (Yang et al., 2013; Du et al., 2017; Jiang et al., 2017; Niu et al., 2017; Zhou et al., 2018). To determine divergent hotspots region in 25 Zingiberaceae species, we compared the Pi values of intergenic regions and coding regions in DnaSP 5.1. The sliding window analysis indicated that the average value of the IR regions was lower than that in the LSC and SSC regions, which showed that most of the variation in LSC and SSC regions (Figure 6). Among the 25 Zingiberaceae species, the average value of nucleotide diversity (Pi) was 0.01257. In LSC and SSC regions, there were five mutational hotspots including *Rps16-trnQ*, *ycf1*, *ndhA*, *psaC*, and *rps12* which exhibited remarkably higher Pi values (>0.03). But in the IR regions, there was no one mutational hotspot that exhibited remarkably higher Pi values (>0.01). The average value of nucleotide diversity (Pi) was 0.00267 among the 14 *Curcuma* species, where there were five mutational hotspots including *matK-trnk*, *Rps16-trnQ*, *petN-psbM*, *rpl32*, *ndhA* and *ycf1* exhibited remarkably higher Pi values (>0.006). By contrast, the Pi values of 25 Zingiberaceae species were higher than those of the 14 *Curcuma* species (Figure 6 and Supplementary Table S8). The mutational hotspots might be adopted as appropriate loci for population genetic and phylogeographic studies.

### Phylogenetic Analyses of the Zingiberaceae Species Based on Chloroplast Genome Sequence Within Zingiberales

**FIGURE 7:**
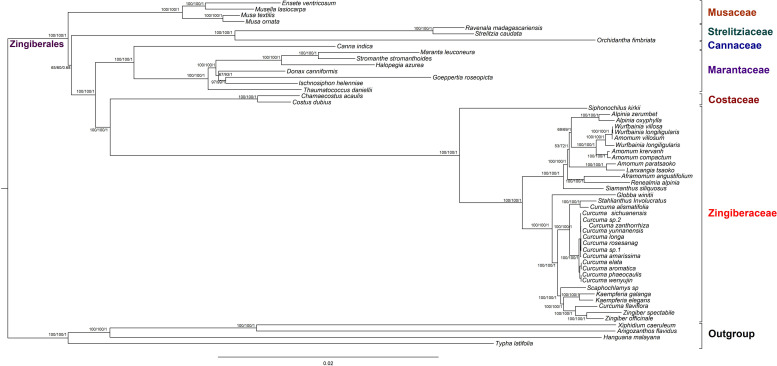
Phylogenetic relationships of 52 Zingiberales species taxa inferred from Maximum Parsimony (MP), Bayesian Inference (BI), and Maximum Likelihood (ML) analyses of protein-coding genes. *Xiphidium caeruleum*, *Anigozanthos flavidus*, *Hanguana malayana*, and *Typha latifolia* was used as the outgroup. Numbers above nodes are support values with MP bootstrap values on the left, ML bootstrap values in the middle, and Bayesian posterior probabilities (PP) values on the right.

To discuss the phylogenetic positions of 56 chloroplast genomes (The species name and GenBank accession numbers could be find in Supplementary Table S9) based on the 79 shared protein coding genes, we used phylogenetic tree to infer the phylogenetic relationships. These chloroplast genomes from seven families within Zingiberales included 35 Zingiberaceae species, four Musaceae species, two Strelitziaceae species, one Lowiaceae species, one Cannaceae species, seven Marantaceae species, two Costaceae species and four other family species as out groups. The topologies of the three dataset (MP, ML, and BI) yielded a similar structure. The seven families of Zingiberales can be classified into seven monophyletic clades (Musaceae, Strelitziaceae, Lowiaceae, Cannaceae, Marantaceae Costaceae, and Zingiberaceae). In addition, Musaceae was the basal group in Zingiberales. Cannaceae was sister to Marantaceae. The Zingiberaceae were gathered into one clade, and *Siphonochilus kirkii* was the first to be isolated (basal taxa). Most species from the same genus were clustered together. The genetic relationship of most of *Wurfbainia* and *Amomum* were very close. Besides that, *Amomum paratsaoko* and *Lanxangia tsaoko* formed a closely relationship. The 14 *Curcuma* species were gathered into one clade except *Curcuma alismatifolia* and *Curcuma flaviflora*. *Curcuma alismatifolia* was initially clustered together with *Stahlianthus Involucratus* and then with other 12 *Curcuma* species. *Curcuma flaviflora* was clustered together with *Zingiber*, and the similar clustering result was measured in previous studies (Cui et al., 2019; Gui et al., 2020). In the ML tree, under 90% were only three nodes with bootstrap values and four nodes in the MP tree (Figure 7). The Musaceae family was the earliest branch and it was sister to the other families in Zingiberales. The 35 Zingiberaceae species clustered for one clade, which was sister to two Costaceae species (*Chamaecostus acaulis* and *Costus dubius*). The tree was cleared to reveal the phylogenetic relationships among Zingiberales species.

## Discussion

In this study, we compared the complete chloroplast genome of 25 Zingiberaceae species, which exhibited a typical quadripartite structures and included 107–116 particular genes composed of 78–85 coding genes, 24–31 tRNAs and 4 rRNAs. In these chloroplast genomes, 155,890–164,101 bp were generated in length, the overall GC contents were between 36.00 and 36.29%. It was obvious that the structure, genome length and organization had a high conservation. Through comparative study, it was found that the intergenic areas was the largest variable regions among the 25 Zingiberaceae chloroplast genomes, and it is a frequent phenomenon in chloroplast genomes (Wicke et al., 2011).

The analysis found that the Ka/Ks and evolutionary rate were low in these Zingiberaceae species as expected. As a common phenomenon in photosynthetic plants, the evolutionary rates of photosynthesis genes (*atpH*, *petL*, *petN*, *psaC*, *psaJ*, *psbM*, *psbT*, *rps2*, and *rps7*) were low. The three genes evolved faster including *atpF* (Subunits of ATP synthase), *rps8* (Small subunit of ribosome) and *ndhC* (Subunits of NADH dehydrogenase). In most cases, the rates of evolution in some genes are species-specific, *clpP* gene is highly conserved plastid-encoding gene, but in some angiosperms is the most variablegene. The plastid gene *Clp* protease complex was sharply different in evolutionary rate (Williams et al., 2019). Unlike most plants, the results indicate that the mean of Ka/Ks of the *clpP* gene in 25 Zingiberaceae species was 0.02. We found two introns were contained in the *clpP* gene, which may lead to the low Ka/Ks. Genes under positive selection usually caused lots of repeating amino acid sequence insertions in varying degrees and may also be closely tied to a near increase in the rate of diversification after they adapted to fit a new ecological environment (Erixon and Oxelman, 2008; Piot et al., 2018).

The interval of the four chloroplast genome regions played an important role in some plant species evolution. In most cases, contraction and expansion of the IR region could result in the production of pseudogenes such as ψ*ycf1*. In addition to *Wurfbainia longiligularis*, the ψ*ycf1* was discovered among the Zingiberaceae species.

Complete chloroplast genomes provide sufficient informative sites for resolving phylogenetic relationships of plant, and have been examined to be effective in the ability of differentiation in lower taxonomic levels (He et al., 2012; Zhang et al., 2016). According to DNA sequences of the nuclear internal transcribed spacer (ITS) and plastid *matK* regions, *Siphonochilus* and *Tamijia* are the basal taxa of the Zingiberaceae family (Kress et al., 2002). But in morphology show that *Alpinieae* is the basal taxa of the Zingiberaceae family (Wood et al., 2000). In our study, we can only speculate that *Siphonochilus* is the basal taxa in Zingiberaceae because of lack of reliable chloroplast genome of *Tamijia*. Besides that, *Curcuma* is paraphyletic with *Stahlianthu* on the basis of the evidence from morphological study. Due to lack of published chloroplast genomes of *Stahlianthu*, our results suggested *Curcuma* had a close relationship with *Stahlianthu* only. The findings were similar to those in previous studies where *Curcuma flaviflora* had a close relation with *Zingiber* species, and they infer that the genes of genetic variations in *Curcuma* has evolved (forest in the wild and heliophyte) in ordet to adapt to the variety of environment (Gui et al., 2020). Besides, *Curcuma* lies dormant in winter, rhizomes fleshy with tuber-bearing roots, and blooms at the rainy season, similar to the drought-enduring plants, and the origination and evolution of *Curcuma* is relate to the drought-enduring plants or not? It is still necessary to need a lot more work to tease out. The MP, ML and BI tree showed a very high level of support in our study. In morphological classification of *Curcuma*, flowers with funnel-shaped corollas, lateral staminodes petaloid, lobes with oblong and ovate and base adnate to labellum and filament. The characteristics of *Zingiber* were valgus lip with 3-lobed, lateral staminodes adnate to labellum, central lobe retuse or cleft at apex and the style is slender, extending beyond anther locules. The morphology of *Curcuma flaviflora* was consistent with the genus of *Curcuma*, although the morphology of *Curcuma flaviflora* and *Zingiber* was different. The distribution range of them overlapped, and the introgressive hybridization may exist extensively. The results showed that *Curcuma* and even Zingiberaceae species may have a complex phylogeny, and more samples and DNA data were necessary to confirm their relationship. To understand their evolutionary history would provide us more useful information to study how to adapt to the environment and raise productivity in plant.

## Materials and Methods

### Plant Material and DNA Extraction

In the study, the fresh leaves of fourteen species of *Curcuma* within Zingiberaceae were provided by Xishuangbanna Tropical Botanical Garden, Chinese Academy of Sciences, and identified by Mr. Jianping Wang. All fresh leaves from 14 samples were desiccated in silica gel and deposited at the herbarium of Sichuan Agriculture University (SICAU). A modified CTAB protocol was used to extract total genomic DNA (Zhang et al., 2017), and the concentration and quality of the extracted DNA were respectively determined by gel electrophoresis and Nanodrop 2000C spectrophotometry.

### Chloroplast Genome Sequencing, Assembly, Mapping, Annotation, and Structure

Following the instruction of library, the purified DNA was used to sequence library construction. Total DNA was sequenced in Illumina HiSeq 2000, Paired-end libraries was read of 150 bp (Gu et al., 2018). Raw sequencing data from the samples were trimmed and filtered by NGSQC Toolkit version 2.3.3 (Patel and Jain, 2012). The SPAdes v3.6.0 was used to *de novo* assembly the matched paired-end reads, and the PRICE (Paired-Read Iterative Contig Extension) was used to repeat the combination of contig and uncompleted *de novo* assembly (Bankevich et al., 2012). In order to check the *de novo* assembly, reads were aligned against the assembled genome using BWA (Li and Durbin, 2009) with default parameters. 15.6% of reads were successfully aligned, but this was sufficient to give a mean coverage of more than 5000×. This mapping enabled us to resolve some short regions of ambiguous sequence in the assembly. The automatic annotator DOGMA was used to annotate the chloroplast genome with BLAST searches against the chloroplast genomes of sibling species. Open reading frames (ORFs) was matched with annotated chloroplast genome, and then the remaining lacking protein evidence ORFs were disregarded (Wyman et al., 2004). The genes were considered potential pseudogenes which contained one or more frameshift mutations or premature stop codons. The online program OGDRAW was used to draw the circular chloroplast genome map and then manually edited (Conant and Wolfe, 2008).

### Codon Usage Analysis

The chloroplast genome of 25 Zingiberaceae species were analyzed for the relative synonymous codon usage (RSCU). When the RSCU value > 1.00, it means the use of a codon is more frequent than expected, and vice versa. The software DAMBE5 was used to obtain the RSCU (Xia, 2013).

### Genome Comparison and Molecular Marker Identification

In order to compare the chloroplast genomes of 25 Zingiberaceae species, we took the reference sequence from *Curcuma sichuanensis*, and used mVISTA in LAGAN mode in pairwise alignments (Frazer et al., 2004). The rates of Ka/Ks was calculated using DnaSP v6.0 (Rozas et al., 2017). Seventy seven protein coding gene from 25 Zingiberaceae were used to calculate evolutionary rate variation (Librado and Rozas, 2009). The nucleotide diversity (Pi) values and sequence polymorphism of Zingiberaceae species were evaluated and the regions of LSC, SSC, and IR were also calculated in DnaSP 5.1 (Rozas et al., 2003).

### Long Repetitive Sequences and Simple Sequence Repeats (SSRs) Analysis

We calculated two types of long repeats: forward (F) and palindromic (P) in the chloroplast genomes. The REPuter was used to detect the size and location of the long repeats (Kurtz et al., 2001). The positions of repeats were identified according to the parameters set as the following conditions: (1) the sequence identity was >90%; (2) the minimum repeat was 30 bp; (3) the Hamming distance was 1. SSRs were detected using the Msatcommander 0.8.2.0 with the following settings: ≥10 for mono-; ≥8 for di-; ≥4 for tri-, tetra-; ≥3 for penta- and hexanucleotide SSRs.

### Phylogenetic Analysis

To determine the phylogenetic relationships and examining the phylogenetic status of Zingiberaceae within Zingiberales, we used MAFFT version 7.0 software to align the 79 shared protein coding gene DNA sequences in default parameter settings (Katoh and Standley, 2013). The Zingiberales species included Zingiberaceae, Musaceae, Strelitziacea, Lowiaceae species, Cannaceae, Marantaceae and Costaceae species with four other species as outgroups. The maximum parsimony (MP) and maximum likelihood (ML) tree were inferred in the software PUAP^∗^ (Swofford and Swofford, 2002). We used Modeltest version 3.7 (Posada and Buckley, 2004) to calculate the best-fit model based on Akaike’s information criterion (AIC). The Bayesian inference (BI) tree was analyzed in MrBayes version 3.1.2 (Ronquist and Huelsenbeck, 2003). A random tree and run for 3,000,000 generations started in Markov chain Monte Carlo (MCMC). Sampling in every 100 generations and 25% of our samples (which the first 2500 trees) were discarded in burnin. The Bayesian posterior probabilities of the nodal supports were inferred and the 50% majority-rule consensus tree was constructed based on the rest of trees.

## Conclusion

In this study, 14 complete chloroplast genome of *Curcuma* were reported, including seven firstly sequenced chloroplast genomes (*C. zanthorrhiza, C. elata, C. yunnanensis, C. alismatifolia, C. amarissima, C. sichuanensis*, and *C. rosesana*) comparatively analyzed with other published genus in the family of Zingiberaceae for the first time. The structure and gene content of chloroplast genome of 25 Zingiberaceae were similarity, and showed highly conserved. The MP, ML, and BI tree showed that 52 Zingiberales species were completely clustered into seven families with high bootstrap values. This study provide the availability of chloroplast genome sequences for improving species identification and phylogenetic research in further study within Zingiberales.

## Data Availability Statement

The assembled chloroplast genome of 14 *Curcuma* was deposited in GenBank: MT395655, MT395645, MT395646, MT395649, MT395651, MT395644, MT395657, MT395653, MT395648, MT395650, MT395652, MT395654, MT395647, and MT395656.

## Author Contributions

RY designed the research and revised the manuscript. HL, YZ, JD, and GG performed the experiments and analyzed the data. HL wrote the manuscript. CD and LZ contributed to the materials and revised the manuscript. All the authors contributed to the article and approved the submitted version.

## Conflict of Interest

The authors declare that the research was conducted in the absence of any commercial or financial relationships that could be construed as a potential conflict of interest.

## Supplementary Material

TABLE S1 Complete chloroplast genomes for 11 Zingiberaceae specie.

TABLE S2 Distribution of genes and Intergenic regions for 11 species in Zingiberaceae.

TABLE S3 The genes having intron in the 25 Zingiberaceae chloroplast genomes.

TABLE S4 Codon content of amino acid and stop codon of 25 Zingiberaceae species.

TABLE S5 The rates of Ka, Ks.

TABLE S6 The comparison of Long repeats among 25 Zingiberaceae species.

TABLE S7 The comparison of SSRs among 25 Zingiberaceae species.

TABLE S8 The Pi value of 14 *Curcuma* species.

TABLE S9 The GenBank accession numbers of 56 species using in phylogenetic analysis.
