# Supplementary material for: The Complete Chloroplast Genome Sequences of 14 Curcuma Species: Insights Into Genome Evolution and Phylogenetic Relationships Within Zingiberales
Source: Front Genet. 2020 Jul 23;11:802. doi: 10.3389/fgene.2020.00802 (PMC7396571; doi:10.3389/fgene.2020.00802)
Supplement: TABLE S1 — Complete chloroplast genomes for 11 Zingiberaceae specie. [file Data_Sheet_1.zip › Supplementary Table 9.DOCX]

**Table S9 The GenBank accession numbers of 56 species using in phylogenetic analysis**

| Number | Species | Genbank number |
| --- | --- | --- |
| 1 | *Zingiber spectabile* | JX088661 |
| 2 | *Zingiber officinale* | MH161428 |
| 3 | *Amomum krervanh* | NC_036935 |
| 4 | *Amomum compactum* | NC_036992 |
| 5 | *Wurfbainia longiligularis* | MK889505 |
| 6 | *Wurfbainia villosa* | MH161418 |
| 7 | *Kaempferia elegans* | NC_040852 |
| 8 | *Kaempferia galanga* | NC_040851 |
| 9 | *Alpinia oxyphylla* | KY985237 |
| 10 | *Stahlianthus Involucratus* | MK262725 |
| 11 | *Lanxangia tsaoko* | MK937808 |
| 12 | *Curcuma zanthorrhiza* | MT395655 |
| 13 | *Curcuma elata* | MT395645 |
| 14 | *Curcuma yunnanensis* | MT395646 |
| 15 | *Curcuma alismatifolia* | MT395649 |
| 16 | *Curcuma amarissima* | MT395651 |
| 17 | *Curcuma sichuanensis* | MT395644 |
| 18 | *Curcuma aromatica* | MT395657 |
| 19 | *Curcuma wenyujin* | MT395653 |
| 20 | *Curcuma flaviflora* | MT395648 |
| 21 | *Curcuma longa* | MT395650 |
| 22 | *Curcuma sp.*1 | MT395652 |
| 23 | *Curcuma* *rosesana* | MT395654 |
| 24 | *Curcuma phaeocaulis* | MT395647 |
| 25 | *Curcuma sp.*2 | MT395656 |
| 26 | *Siphonochilus kirkii* | MH603442 |
| 27 | *Alpinia zerumbet* | JX088668 |
| 28 | *Wurfbainia longiligularis* | NC_044774 |
| 29 | *Amomum villosum* | MK389642 |
| 30 | *Lanxangia paratsaoko* | MH423780 |
| 31 | *Aframomum angustifolium* | MH603398 |
| 32 | *Renealmia alpinia* | MH603438 |
| 33 | *Siamanthus siliquosus* | MH603441 |
| 34 | *Globba winitii* | MH603419 |
| 35 | *Scaphochlamys sp.* | MH603440 |
| 36 | *Ensete ventricosum* | MH603417 |
| 37 | *Musella lasiocarpa* | NC_035637 |
| 38 | *Musa textilis* | NC_022926 |
| 39 | *Musa ornata* | NC_042874 |
| 40 | *Ravenala madagascariensis* | KF601568 |
| 41 | *Strelitzia caudata* | MH603443 |
| 42 | *Orchidantha fimbriata* | KF601569 |
| 43 | *Canna indica* | KF601570 |
| 44 | *Maranta leuconeura* | KF601571 |
| 45 | *Stromanthe stromanthoides* | MH603445 |
| 46 | *Halopegia azurea* | MH603421 |
| 47 | *Donax canniformis* | MH603414 |
| 48 | *Goeppertia roseopicta* | MH603420 |
| 49 | *Ischnosiphon helenniae* | MH603427 |
| 50 | *Thaumatococcus daniellii* | KF601575 |
| 51 | *Chamaecostus acaulis* | MH603404 |
| 52 | *Costus dubius* | MH603406 |
| 53 | *Xiphidium caeruleum* | JX088669 |
| 54 | *Anigozanthos flavidus* | NC_040160 |
| 55 | *Hanguana malayana* | NC_029962 |
| 56 | *Typha latifolia* | NC_013823 |
